# Synergism between obesity and HFpEF on neutrophils phenotype and its regulation by adipose tissue‐molecules and SGLT2i dapagliflozin

**DOI:** 10.1111/jcmm.17466

**Published:** 2022-07-11

**Authors:** Cristina Almengló, Xiaoran Fu, María Teresa Flores‐Arias, Ángel L. Fernández, Juan E. Viñuela, José M. Martínez‐Cereijo, Darío Durán, Moisés Rodríguez‐Mañero, José R. González‐Juanatey, Sonia Eiras

**Affiliations:** ^1^ Cardiology Group Health Research Institute Santiago de Compostela Spain; ^2^ Translational Cardiology Group Health Research Institute Santiago de Compostela Spain; ^3^ Photonics4 Life Research Group, Applied Physics Department, Facultade de Física and Facultade de Óptica e Optometría Universidade de Santiago de Compostela Santiago de Compostela Spain; ^4^ Heart Surgery Department University Hospital of Santiago de Compostela Santiago de Compostela Spain; ^5^ CIBERCV Madrid Madrid Spain; ^6^ Immunology Laboratory Health Research Institute Santiago de Compostela Spain; ^7^ Cardiovascular Department University Hospital of Santiago de Compostela Santiago de Compostela Spain

**Keywords:** adiposity, cardiovascular disease, neutrophils

## Abstract

The adiposity invokes innate immune activity, coronary microvascular dysfunction and consequently heart failure preserved ejection fraction (HFpEF). Our aim was to study the neutrophils profile on obesity and cardiovascular disease and its regulation by adipose tissue‐secretome and dapagliflozin. We have isolated neutrophils from patients undergoing open heart surgery (19 women and 51 men). Its migration activity was performed with culture‐transwell, transcriptional studies of proteolytic enzymes, adhesion molecules or receptors were analysed by real‐time PCR and proteomics (from 20 patients) analysis by TripleTOF mass spectrometer. Differentiated HL‐60 (dHL‐60) was used as a preclinical model on microfluidic for endothelial cells attaching assays and genes regulation with epicardial and subcutaneous fat secretomes from patients (3 women and 9 men) or dapagliflozin 1–10 μM treatments. The transcriptional and proteomics studies have determined higher levels of adhesion molecules in neutrophils from patients with obesity. The adhesion molecule CD11b levels were higher in those patients with the combined obesity and HFpEF factors (1.70 ± 0.06 a.u. without obesity, 1.72 ± 0.04 a.u. obesity or HFpEF without obesity and 1.79 ± 0.08 a.u. obesity and HFpEF; *p* < .01). While fat‐secretome induces its upregulation, dapagliflozin can modulated it. Because CD11b upregulation is associated with higher neutrophils migration and adhesion into endothelial cells, dapagliflozin might modulate this mechanism on patients with obesity and HFpEF.

## 1 INTRODUCTION

Obesity contributes to cardiovascular disease and development of worse outcomes.^1^ The excessive fat accumulation has hemodynamic, inflammatory, mechanical and neurohormonal effects. All of these mechanisms participate in heart failure preserved ejection fraction (HFpEF).^2^ Obesity is a chronic low‐grade inflammatory state caused, in part, by the released pro‐inflammatory cytokines of adipose tissue, which can regulate the neutrophils phenotype and activity.^3^ These immune cells are the most abundant granulocytes that participates in the inflammation‐resolution and host‐defence mechanism.^4^ They have a functional and phenotypic plasticity. The chemokine receptors of the neutrophils, such as CXCR‐4 or CXCR‐2, are the key master of their homeostasis. But the downregulation of CXCR‐4 and upregulation of CXCR‐2 increases their mobilization from the bone marrow to the blood^5^ or to adipose tissue recruitment on obesity and diabetes.^6^ This last process is also produced by the upregulation of the adhesion molecules, such as integrin alpha M (ITGAM), named also cluster of differentiation molecule 11B (CD11B). But the increment of this molecule also participates on neutrophils migration and infiltration into atrium^7^ and on circulating monocytes indicates an adipose tissue‐metabolic dysfunction.^8^ The neutrophils infiltration into adipose tissue promotes the release of pro‐inflammatory cytokines and enhances the inflammation on this tissue.^9^ Some of the neutrophils‐proteolytic enzymes, myeloperoxidase (MPO) or elastase, might start this process.^10^ Thus, plasma MPO levels, which are indicators of circulating neutrophils activity, are raised in patients with severe obesity,^11^ cardiovascular risk and poor prognosis.^10^ Although the excessive neutrophils migration into tissues contributes to inflammatory diseases, the absence of neutrophils‐adhesion molecules might also reduce their migration activity into inflamed tissues and enhance the infections complications after surgery. In this line, a study suggested a higher probability of post‐surgery infection in morbidly obese patients who had a downregulation of the adhesion molecule L‐selectin, named also CD62 antigen‐like family member L (CD62L) without changes on CD11B or the cluster of differentiation 16 (CD16).^12^ Other molecule, which is upregulated in these patients, is the metalloproteinase 9 (MMP9).^13^ This protein and lipocalin‐2 (LNC‐2), also named neutrophil gelatinase‐associated lipocalin (NGAL), are involved in the weaken endothelium junctions^14^ and associated with severity of coronary artery disease (CAD).^15^ There is a clear network evidence between neutrophils stage and obesity‐related cardiovascular disease progression.^16^ However, the knowledge of the main upregulated neutrophils’s molecules on each obesity‐related cardiovascular disease process might be useful as future preventive therapeutic target since some of them are not modulated by diet or exercise.^17^ The inflammatory phenotype, that is associated with obesity and HFpEF^18^ might suggest a differential neutrophils phenotype on these patients. Recent drugs that inhibit the sodium‐glucose cotransporter 2 (SGLT2i), with effects on attenuation of visceral adiposity^19^ and cardiac inflammation via blunting inflammasome^20^, ^21^ have demonstrated their benefits on HFpEF.^22^ Even, some authors have described the role of SGLT2i on neutrophils recruitment and neutrophils extracellular traps (NETs) formation,^23^ which is dependent on glycolysis metabolism. Our main objective was to study the neutrophils phenotype on patients with obesity and cardiovascular disease, specifically in HFpEF, its regulation by adipose tissue‐released molecules and the SGLT2i, dapagliflozin.

## 2 METHODS

### 2.1 Patients' samples and regulatory approval

We have included preoperative and peripheral blood samples from 70 patients and epicardial and subcutaneous white adipose tissue from 12 patients undergoing open heart surgery after signing the informed consent. All the patients with previous inflammatory disease were excluded from the study, following the approved protocol by Galician Clinical Committee (protocol code 2019_439, 22/10/2019) and the Declaration of Helsinki rules.

#### 2.1.1 Neutrophil's isolation

Fasting (for 14 h) blood samples were collected by venepuncture into lithium heparin‐coated vacutainers before surgery procedure, transferred and processed into the laboratory within the first hour. Neutrophils were isolated by single‐step centrifugation of whole blood onto Polymorphprep (Proteogenix, Schiltigheim, France) as the manufacturer's recommendation. Briefly, whole blood was tipped on Polymorphprep at a ratio (1: 3) and centrifuged at 500g for 35 min. The granulocytes layer was carefully removed and resuspended in RPMI 1640 media supplemented with 25 mM HEPES (Lonza Biologics, Porriño, Spain). Then, cells were washed to remove any remaining Polymorphprep and resuspended in media. Neutrophil's size and number were determined by Sceptre™ 2.0 Cell Counter (Millipore®, Merck Life Science S.L.U., Madrid, Spain). Four hundred thousand cells with a specific size between 9 and 12 μm were used for proteomics and migration assays. The rest of neutrophils was used for RNA analysis.

#### 2.1.2 Neutrophil's RNA expression levels

Neutrophils were lysed and RNA was isolated, following the manufacturer's protocol, with AllPrep DNA/RNA/Protein Mini Kit (Qiagen, Hilden, Germany). After retro‐transcription, using the Maxima First Strand cDNA Synthesis Kit (Thermo Fisher Scientific, Waltham, MA, USA), 1 μl of cDNA was used for amplifying MPO (F: 5′‐TTTGACAACCTGCACGATGAC‐3′; R:5′‐CGGTTGTGCTCCCGAAGTAA‐3′), CD16 (F:5′‐CCTCCTGTCTAGTCGGTTTGG‐3′;R:5′TCGAGCACCCTGTACCATTGA‐3′), olfatomedin‐4 (OLFM4) (F:5′‐AGCTCTTTCCCAGGTGTTGA‐3′; R: 5′‐AAGCGTTCCACTCTGTCCAC‐3′), CXCR2 (F:5′‐‐3′; R: 5′‐AGTGTGCCCTGAAGAAGAGC‐3′), NGAL (F:5′‐CAGGACTCCACCTCAGACCT‐3′; R: 5′‐CCAGGCCTACCACATACCAC‐3′), intercellular adhesion molecule 1 (ICAM) (F:5′‐GAGATCACCATGGAGCGGAAT‐3′; R: 5′‐ACTGTGGGGTTCAACCTCTG‐3′), MMP9 (F:5′‐ATCCAGTTTGGTGTCGCGGAGC‐3′; R: 5′‐GAAGGGGAAGACGCACAGCT‐3′) S100 calcium binding protein A9 (S100A9) (F:5′‐CTCAAGAAGGAGAATAAGAATGAAAAGG‐3′; R: 5 ′‐TCAGCTGCTTGTCTGCATTTG‐3′),CD62L (F:5′‐AAACCCATGAACTGGCAAAG‐3′; R: 5′‐CGCAGTCCTCCTTGTTCTTC‐3′), CXCR4 (F:5′‐CACTTCAGATAACTACACCG‐3′; R:5′‐ATCCAGACGCCAACATAGAC‐3′), Lactotransferrin (LTF) (F:5′‐CTCCAGACCGCAGACATGAA‐3′; R:5′‐CTGGGAGGAGAAGGCACATT‐3′), neutrophil defensin 3 (DEFA3) (F:5′‐TCCCAGAAGTGGTTGTTTCC‐3′; R:5′‐CAGAATGCCCAGAGTCTTCC‐3′), CD11B (F:5′‐CAGCCTTTGACCTTATGTCATGG‐3′; R:5′‐CCTGTGCTGTAGTCGCACT‐3′), and b‐actin (ACTB) using the FastStart SYBR Green Master (Hoffman‐La Roche, Basilea, Switzerland) at 40 cycles (95°C for 30 s, 58°C for 60 s and 72°C for 60 s) in a QuantStudio 3 (Thermo Fisher Scientific, Walthman, MA, USA). The cycle threshold (Ct) values of the genes were normalized by the Ct values of ACTB. The expression levels were represented as 2^(ACTB/gene)^ algorithm and arbitrary units (a.u.).

#### 2.1.3 Western blot

CD11b protein detection in neutrophils from patients with cardiovascular disease was performed with the extracted protein according to kit manufacturer's instructions (Qiagen, All prep. DNA/RNA/protein). Lysed proteins were resolved on 10% sodium dodecyl sulphate polyacrylamide gel electrophoresis (SDS‐PAGE) and electro blotted onto a nitrocellulose membrane (Trans‐Blot Transfer Medium 0.45 μm, Merck Millipore, Darmstadt, Germany). Membranes were subsequently blocked with 5% bovine serum albumin diluted in Tris‐buffered saline with 0.1% Tween 20 (TBS‐T) and incubated overnight at 4°C or 4 h at room temperature with appropriate antibodies against CD11b (dilution 1:1000; clone 238439, cat. no. MA16992, R&D Systems) and anti‐β‐actin (dilution 1:5000; cat. no. sc‐69879, Santa Cruz Biotechnology, Heidelberg, Germany), Used as secondary antibody alexa fluor 488‐rabitt anti‐mouse. Immunofluorescence bands were detected with Chemidoc MP Imager (BioRad Laboratories, Madrid, Spain).

#### 2.1.4 Neutrophils' proteomics approach

Neutrophils from consecutively selected 20 patients (10 with and 10 without obesity) were used for proteomics analysis. Proteins were isolated with AllPrep DNA/RNA/Protein Mini Kit (Qiagen, Hilden, Germany), then, were separated by SDS‐polyacrylamide gel (PAGE) and Coomassie stanning. Identification of proteins were performed as it was described.^24^ After trypsin digestion following standard procedure with minor modifications,^25^ proteins were identified by mass spectrometry. Digested peptides were separated using Reverse Phase Chromatography. Gradient was developed using a micro liquid chromatography system (Eksigent Technologies nanoLC 400, SCIEX Foster City, CA) coupled to high‐speed Triple TOF 6600 mass spectrometers (SCIEX) with a micro flow source. The analytical column used was a silica‐based reversed phase column YMC‐TRIART C18 150 × 0.30 mm, 3 mm particle size and 120 Å pore size (YMC Technologies, Teknokroma). The trap column was a YMC‐TRIART C18 (YMC Technologies, Teknokroma) with a 3 mm particle size and 120 Å pore size, switched on‐line with the analytical column. The loading pump delivered a solution of 0.1% formic acid in water at 10 μl/min. The micro‐pump provided a flow‐rate of 5 μl/min and was operated under gradient elution conditions, using 0.1% formic acid in water as mobile phase A, and 0.1% formic acid in acetonitrile as mobile phase B. Peptides were separated using a 90 min gradient ranging from 2% to 90% mobile phase B (mobile phase A: 2% acetonitrile, 0.1% formic acid; mobile phase B: 100% acetonitrile, 0.1% formic acid). Injection volume was 4 μl. Data acquisition was carried out in a Triple TOF 6600 System (SCIEX, Foster City, CA, USA) using a data‐dependent workflow. Source and interface conditions were as follows: ion spray voltage floating (ISVF) 5500 V, curtain gas (CUR) 25, collision energy (CE) 10 and ion source gas 1 (GS1) 25. Instrument was operated with Analyst TF 1.7.1 software (SCIEX, Foster City, CA). Switching criteria was set to ions greater than mass to charge ratio (*m*/*z*) 350 and smaller than *m*/*z* 1400 with charge state of 2–5, mass tolerance 250 ppm and an abundance threshold of more than 200 counts (cps). Former target ions were excluded for 15 s. Instrument was automatically calibrated every 4 h using tryptic peptides from Beta Galactosidase as external calibrant.

After MS/MS analysis, data files were processed using Protein Pilot TM 5.0.1 software from Sciex which uses the algorithm Paragon TM for database search and Progroup TM for data grouping. Data were searched using a Human specific Uniprot database. False discovery rate was performed using a non‐lineal fitting method displaying only those results that reported a 1% global false discovery rate or better.^26^, ^27^ The mass spectrometry proteomics data have been deposited to the ProteomeXchange Consortium via the PRIDE partner repository with the dataset identifier PXD032826.

#### 2.1.5 Neutrophil's migration

Neutrophils (400,000 cells) from patients with left ventricle ejection fraction (LVEF) ≥ 50% (*n* = 47) were seeded into transwell with a pore size of 6 μm (Merck Life Science S.L.U., Madrid, Spain). Migrated cells through transwell were determined after being treated with or without complement component 5a (C5a), at a final concentration of 11 nM for 90 min. This complement contributes to the migratory phenotype of neutrophils,^28^ inflammation and insulin resistance on obesity.^29^ Migrated cells were detached with EDTA (0.05 M) for 15 min at 4°C and afterwards migrated and non‐migrated neutrophils were collected and centrifuged at 500g for 5 min. Finally, the neutrophils pellet was lysed and frozen at −80°C. The frozen lysed neutrophils were thawed at room temperature and DNA was quantified using CyQUANT® GR dye (Thermo Fisher Scientific, Waltham, MA, USA), following the manufacturer's instructions. After that, fluorescence that represents also migrated and no migrated neutrophils, was recorded at an excitation/emission wavelength (485/525 nm) with (FLUOstar OPTIMA). The results were expressed as a percentage of migrated regarding total cells in relative fluorescence units (RFU).

### 2.2 HL‐60 cell line

HL‐60 cell line was differentiated into neutrophils (dHL‐60) with dimethylsulphoxide (DMSO) at (1.26%) for 6 days. Afterwards, 400,000 dHL‐60 cells/well were cultured in 24 wells‐plates treated with N‐formylmethionyl‐leucyl‐phenylalanine (fMLP) (Merck Life Science S.L.U., Madrid, Spain) at 1uM, a known up‐regulator of CD11B,^30^ for 90 min.

### 2.3 Human umbilical vein endothelial cells (HUVEC) primary culture

HUVEC were isolated from freshly obtained human umbilical cords donated after local ethics committee approval and informed consent from mothers, following the Declaration of Helsinki and method previously described.^31^ Cells were cultured on 0.2% (w/v) gelatine (Merck Life Science S.L.U., Madrid, Spain) pre‐coated flasks or dishes (Corning, New York, NY, USA) and grown in complete Endothelial Growth Medium‐2 (EGM‐2) (Lonza Biologics, Porriño, Spain), containing 2% foetal bovine serum (FBS) between other components, in a humidity‐saturated atmosphere with 5% CO_2_ at 37°C. Cells for the experiments were used between the second and seventh passages.

### 2.4 Microfluidic model

The fluidic device was a concave Y‐shaped channel made from polydimethylsiloxane (PDMS, Sylgard 184 from Dow Corning) following the method previously described.^32^ For the experiments, HUVEC at confluence were detached with trypsin (0.25% in Hank's balanced salt solution with 1 mM EDTA (Thermo Fisher Scientific, Waltham, MA, USA) and seeded in fibronectin (5 μg/ml in 0.02% gelatine; Gibco®) the pre‐coated PDMS channels at a concentration of 1.5 × 10^6^ cells/ml and maintained overnight until the start of the fluidic experiment. Perfusion started at 0.5 ml/min with EGM‐2 medium to avoid damage to the monolayer, velocity was doubled every hour until reach a flow‐rate of 6 ml/min, that was maintained for 4 h. Differentiated HL‐60 with or without CD11b upregulation were labelled with calcein‐AM (Invitrogen®, Thermo Fisher Scientific, Waltham, MA, USA) at a 1 μM concentration for 20 min. Then, cells were rinsed twice to remove unlabelled calcein‐AM and 5 × 10^5^ were incorporated to the flow under sterile conditions when the flow‐rate reached the 6 ml/min. At the end of the experiment, the flow was stopped, and the channels were washed with saline solution. Fluorescent and contrast phase images (Zeiss Axio Vert.A1, Zeiss, Oberkochen, Germany) were obtained to calculate the number of adhered neutrophils to the endothelial monolayer (ImageJ software).^33^

### 2.5 Supernatants of epicardial and subcutaneous fat and dapagliflozin on CD11b of differentiated HL‐60 (dHL‐60)

After testing the positive and adhesion activity of treated dHL‐60, 400,000 cells/well were cultured in 24 wells‐plates and treated with fMLP or conditioned medium of epicardial fat or subcutaneous fat (0.2 ml). This conditioned medium was collected from 12 consecutive patients with and without obesity undergoing open heart surgery. Fat biopsies were split into 100 mg pieces and embedded with M199 medium (Lonza Biologics, Porriño, Spain) (100 mg/0.5 ml) for 24 h. Afterwards, the cells were removed by centrifuging and supernatant was stored at −80°C until be used. At the end, RNA expression levels of CD11B were determined as it was described above.

Pre‐treatment of dHL‐60 with dapagliflozin at 1 μM or 10 μM for 2 h was performed for studying its effects on CD11B expression levels after fMLP stimulation.

### 2.6 Statistical analysis

Continues data were tested for normality using the Shapiro–Wilk test, and for homoscedasticity with the Levene's test and expressed by mean ± standard deviation (SD). Categorical variables were represented as percentage and differences between them were analysed by Pearson's chi‐squared test. If the groups differed regarding more than two variables, logistic regression was applied for discriminate the best variable associated with mRNA expression levels. The regulation of neutrophils genes was analysed by comparison among groups (non‐obesity, obesity without HFpEF or HFpEF without obesity and obesity with HFpEF) with anova and post hoc Bonferroni test. Comparison between control with or without fMLP and fat‐conditioned medium or dapagliflozin was carried out using the paired Student's *t* test in the normal distribution data and the Wilcoxon signed rank test in the skewed data. Statistical Package for Social Science (SPSS) for Windows, version 15.0 (software SPSS Inc) package, was used for all statistical analyses. Statistical significance was defined as *p* < .05.

## 3 RESULTS

### 3.1 Transcriptional and proteomics approaches on neutrophils regarding obesity

**FIGURE 1:**
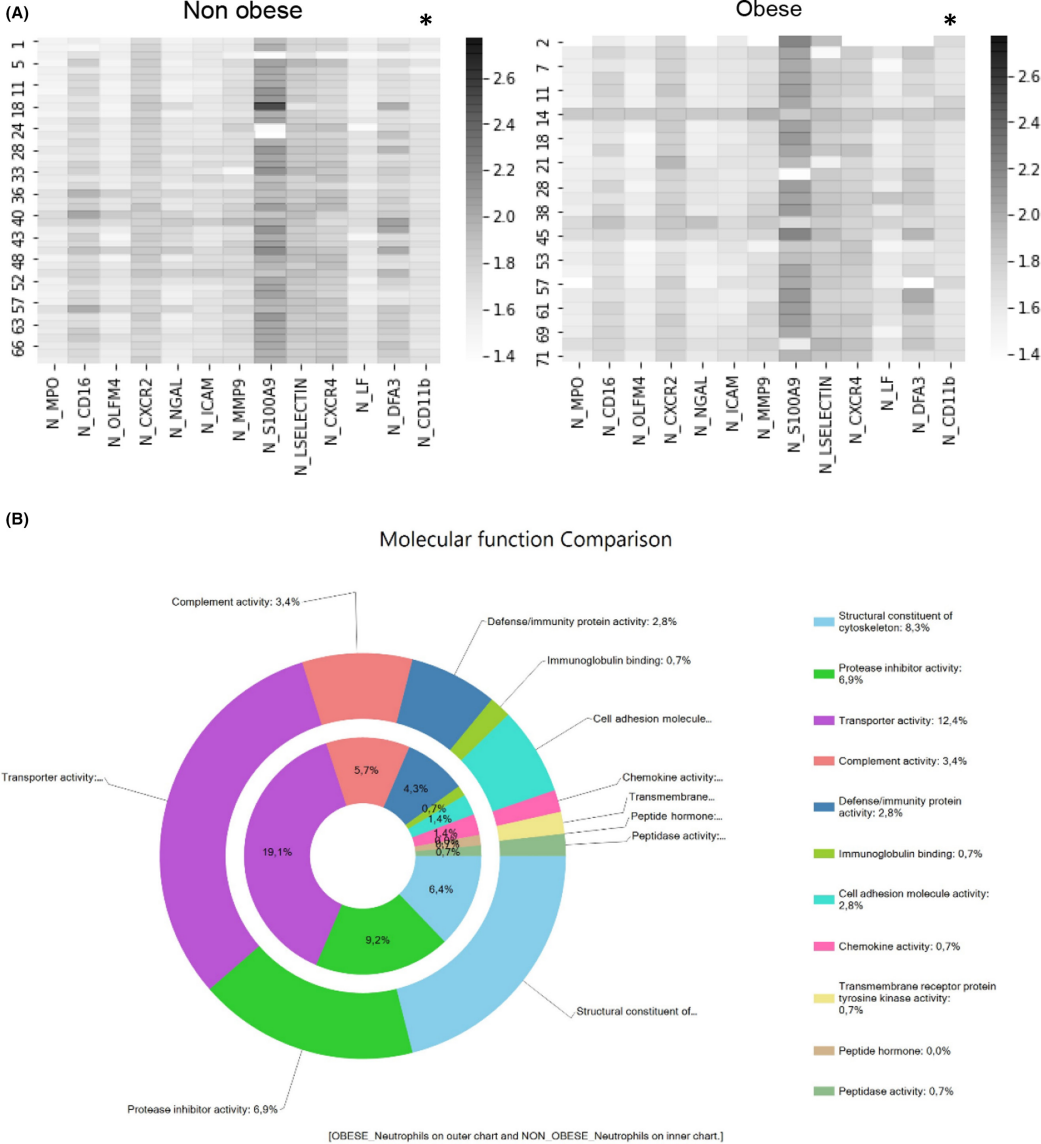
Transcriptional profiles of neutrophilic markers from patients without (NOB) and with obesity (OB) (A). Heatmaps represents the mRNA expression levels of the neutrophil's markers. Differential genes expression with statistical significance between groups were described in CD11b regarding obesity (*p < .05). Proteomics approach: identified proteins on neutrophils from patients without or with obesity. Functional classification of neutrophil's proteins from patients without or with obesity (B). The ring charts show a greater number of proteins contained in azurophil granules and involved in cell adhesion in obese patients compared with non‐obese patients

Patients were classified according to obesity. The main differential risk factors and genes expression levels on neutrophils are represented on Table 1. Although, proteases enzymes, adhesion molecules and receptors were analysed, only CD11B was upregulated on patients with cardiovascular disease and obesity (1.74 ± 0.06 a.u. vs. 1.71 ± 0.05 a.u.; *p* < .05) (Figure 1A). We have selected twenty consecutive patients with and without obesity for neutrophils's proteomic analysis without other clinical variable different between them (Table S1). Two hundred ninety‐four proteins were identified in total, of whom 130 were common in neutrophils from patients with and without obesity, 66 only in patients without obesity and 98 in those with obesity. Higher number of the identified proteins with a usual localization on azurophilic granules and involved on cellular adhesion and tyrosine kinase signalling were detected in patients with obesity (Figure 1B). However, most of the differential proteins were detected in one or two patients without reach the statistical significance. Only five proteins, ceruloplasmin (CERU), fetuin A (FETUA), succinate‐‐CoA ligase [ADP/GDP‐forming] subunit alpha (SUCA), non‐secretory ribonuclease (RNAS2) and eosinophil cationic protein (ECP), named also RNAS3, were differential more identified in obesity than non‐obesity (Table S2) with statistical significance (*p* < .05). These two last proteins were not identified in patients without obesity. The STRING analysis determined that these proteins were mainly localized on vesicles and interact with metabolism‐involved molecules (Figure S1).

**TABLE 1:** Clinical characteristics regarding obesity

	Non‐obesity (*n* = 45)	Obesity (*n* = 25)	*p*
Age	67 (7)	70 (7)	.742
BMI (kg/m^2^)	26 (2)	33 (5)	**.000**
Gender (female/male)	14/31	5/20	.317
CAD (no/yes)	26/19	7/18	**.017**
T2DM (no/yes)	32/13	16/9	.539
AHT (no/yes)	17/28	4/21	.057
DLP (no/yes)	9/36	4/21	.680
KF (no/yes)	22/3	44/1	.267
HF (no/yes)	21/23	11/14	.765
LVEF > 50% (no/yes)	15/30	7/18	.645
AF (no/yes)	41/4	20/5	.183
Neutrophils (10^3^/μl)	4.02 (1.48)	4.43 (1.67)	.535
Monocytes (10^3^/μl)	0.46 (0.17)	0.44 (0.15)	.550
N‐MPO	1.62 (0.07)	1.64 (0.09)	.340
N‐CD16	1.83 (0.12)	1.79 (0.09)	.198
N‐OLFM4	1.64 (0.10)	1.61 (0.11)	.257
N‐CXCR2	1.88 (0.05)	1.88 (0.07)	.928
N‐NGAL	1.68 (0.11)	1.65 (0.12)	.318
N‐ICAM	1.71 (0.08)	1.69 (0.07)	.278
N‐MMP9	1.78(0.09)	1.79 (0.13)	.624
N‐S100A9	2.13 (0.21)	2.11 (0.23)	.806
N‐L‐SEL	1.92 (0.07)	1.91 (0.13)	.400
N‐CXCR4	1.90 (0.10)	1.90 (0.05)	.969
N‐LF	1.66 (0.08)	1.68 (0.11)	.287
N‐DEFA3	1.86 (0.14)	1.88 (0.11)	.616
N‐CD11B	1.71 (0.05)	1.74 (0.06)	**.013**
N‐CD88	1.79 (0.09)	1.73 (0.09)	.077

*Notes*: All genes are expressed in arbitrary units (a.u.); Statistical significance: *p* < .05.

Abbreviations: AHT, arterial hypertension; AF, atrial fibrillation; BMI, body mass index; CAD, coronary artery disease; DLP, dyslipidaemia; HF, heart failure; KF, kidney failure; N‐, neutrophils; T2DM, type 2 diabetes mellitus.

### 3.2 Differential genes expression levels on neutrophils regarding obesity and HFpEF

**FIGURE 2:**
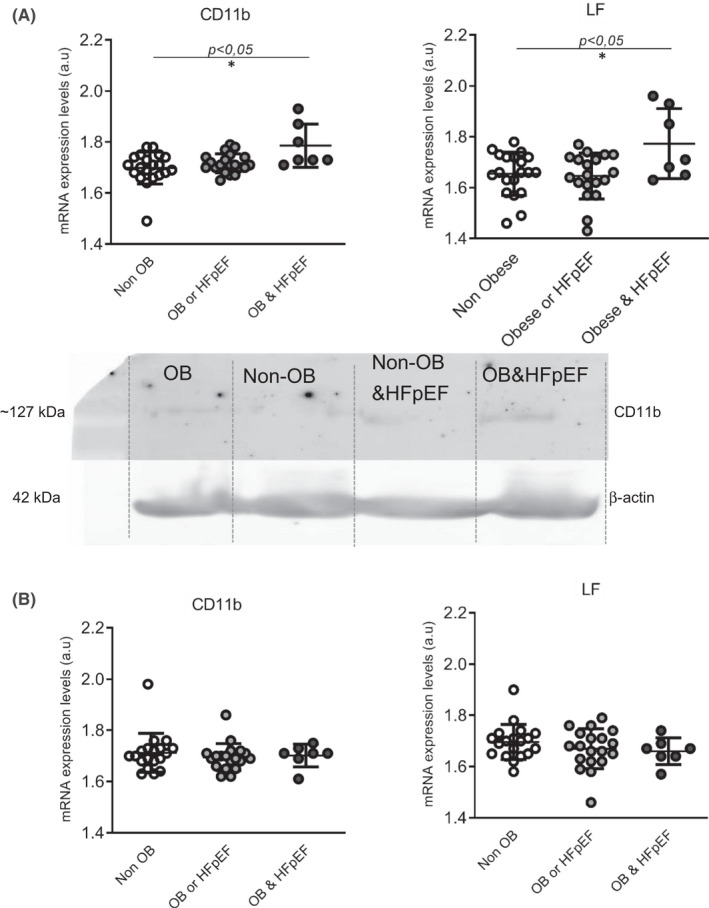
A mRNA expression levels of CD11b and LF, in neutrophils (A) and monocytes (B) after classifying patients according (without obesity, non‐obese; with obesity or HFpEF without obesity and obesity with HFpEF). Dot plots represent mean ± standard deviation (SD) and individual values. Western blot for CD11b and b‐Actin protein detection is represented below dot plot for CD11B mRNA levels. The statistical significance among patients was analysed by ANOVAs and post hoc test results are depicted as *p < .05

We have selected patients with left ventricle ejection fraction (LVEF) >50%. Afterwards, we classified patients according obesity presence or/and HFpEF. The clinical characteristics are represented on Table 2. The main differences regarding CD11B were emphasized in patients with obesity and HFpEF (1.70 ± 0.06 a.u. without obesity, 1.72 ± 0.04 a.u. obesity or HFpEF without obesity and 1.79 ± 0.08 a.u. obesity and HFpEF; *p* < .01). These differences were not found on monocytes from the same patients (Figure 2).

**TABLE 2:** Clinical characteristics regarding obesity and HFpEF

	Non‐obesity non HFpEF (*n* = 20)	Obesity or HFpEF without obesity (*n* = 20)	Obesity and HFpEF (*n* = 7)	*p*
Age	69 (8)	69 (7)	67 (9)	.826
BMI (kg/m^2^)	26.60 (2.7)	30.78 (6.6)	33.00 (2.19)	**.004**
Gender (female/male)	6/14	7/13	3/4	.82
CAD (no/yes)	15/5	11/9	1/6	**.019**
T2DM (no/yes)	17/3	16/4	5/2	.729
AHT (no/yes)	7/13	7/13	2/5	.947
DLP (no/yes)	6/14	2/18	1/6	.258
KF (no/yes)	20/0	19/1	6/1	.267
HF (no/yes)	20/0	11/9	0/7	**.000**
AF (no/yes)	18/2	17/3	6/1	.886
%C5a	31.20 (13.60)	36.86 (20.07)	15.98 (12.69)	**.024**
%Basal	30.54 (17.39)	33.31 (17.63)	26.18 (19.91)	.655
Basal_C5a	1.15 (0.80)	1.10 (0.88)	1.61 (0.45)	.126
N‐MPO	1.61 (0.07)	1.61 (0.06)	1.68 (0.14)	.109
N‐CD16	1.81 (0.10)	1.80 (0.09)	1.85 (0.08)	.354
N‐OLFM4	1.62 (0.07)	1.61 (0.09)	1.67 (0.14)	.350
N‐CXCR2	1.88 (0.05)	1.88 (0.07)	1.90 (0.04)	.693
N‐NGAL	1.66 (0.11)	1.66 (0.11)	1.75 (0.15)	.200
N‐ICAM	1.72 (0.08)	1.68 (0.05)	1.73 (0.10)	.196
N‐MMP9	1.76 (0.11)	1.77 (0.12)	1.88 (0.12)	.071
N‐S100A9	2.12 (0.19)	2.15 (0.28)	2.12 (0.18)	.911
N‐L‐SEL	1.93 (0.08)	1.88 (0.15)	1.95 (0.05)	.223
N‐CXCR4	1.89 (0.12)	1.89 (0.07)	1.89 (0.06)	.983
N‐LF	1.65 (0.08)	1.64 (0.09)	1.77 (0.14)	**.011**
N‐DEFA3	1.84 (0.15)	1.90 (0.13)	1.86 (0.08)	.457
N‐CD11B	1.70 (0.06)	1.72 (0.04)	1.79 (0.08)	**.004**
N‐CD88	1.79 (0.09)	1.75 (0.06)	1.79 (0.07)	.485
M‐MPO	1.62 (0.09)	1.61 (0.06)	1.59 (0.05)	.699
M‐CD16	1.84 (0.14)	1.76 (0.10)	1.78 (0.07)	.071
M‐OLFM4	1.65 (0.12)	1.62 (0.08)	1.60 (0.06)	.382
M‐CXCR2	1.83 (0.15)	1.75 (0.09)	1.80 (0.09)	.126
M‐NGAL	1.67 (0.13)	1.64 (0.10)	1.62 (0.06)	.544
M‐ICAM	1.68 (0.09)	1.64 (0.05)	1.68 (0.05)	.175
M‐MMP9	1.77 (0.11)	1.70 (0.12)	1.76 (0.03)	.156
M‐S100A9	2.15 (0.20)	2.00 (0.24)	2.08 (0.19)	.118
M‐L‐SEL	1.95 (0.15)	1.86 (0.06)	1.90 (0.07)	**.043**
M‐CXCR4	1.88 (0.16)	1.78 (0.15)	1.84 (0.03)	.094
M‐LF	1.69 (0.07)	1.67 (0.08)	1.66 (0.05)	.404
M‐DEFA3	1.89 (0.14)	1.86 (0.06)	1.90 (0.11)	.518
M‐CD11B	1.71 (0.08)	1.70 (0.05)	1.70 (0.04)	.703
M‐CD88	1.79 (0.13)	1.72 (0.09)	1.75 (0.07)	.182

*Notes*: All genes are expressed in arbitrary units (a.u.). Statistical significance: *p* < .05.

Abbreviations: AF, atrial fibrillation; AHT, arterial hypertension; BMI, body mass index; CAD, coronary artery disease; DLP, dyslipidaemia; HF, heart failure; KF, kidney failure; M‐, monocytes; N‐, neutrophils; T2DM, type 2 diabetes mellitus.

### 3.3 Migratory activity of neutrophils

**FIGURE 3:**
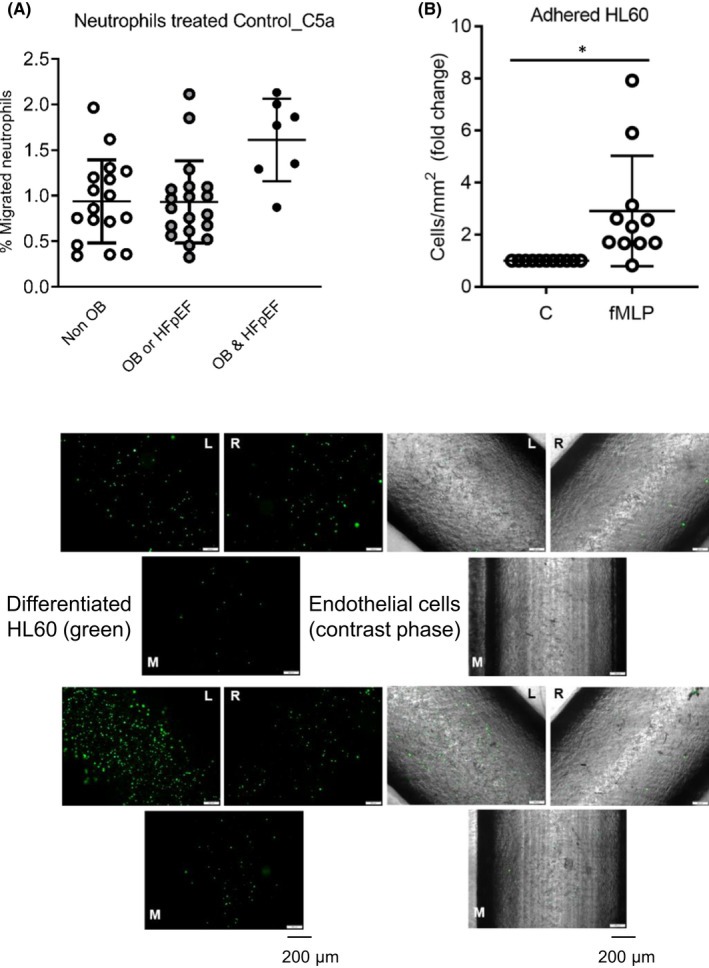
Neutrophil's migration (A). The migratory capacity of neutrophils (ratio between basal migration/C5a treatment. Dot plots represent individual values and mean ± standard deviation (SD). Microfluidic 3D assay and differentiated HL60 with an CD11b upregulation (B). Differentiated or not HL60 at 5 × 10^5^ cells/ml were included in a microchannel system under flow at 6 ml/min flow‐rate during 4 h. After this time, adhered neutrophils into the endothelial cells‐impregnated walls were visualized and counted. Differentiated HL60 with upregulated CD11b were more attached into endothelial cells than those with lower CD11b levels (2.9 ± 0.6‐fold change; *p < .05). C = basal control, (cells in basal medium) and fMLP = positive control. Representative images of attached neutrophils into endothelial cells‐impregnated microchannels. Non‐differentiated HL60 (A) showed less adhesion to endothelial monolayer than the differentiated HL‐60 with an upregulation of CD11b (B). Differentiated HL60 were stained with calcein and showed in green (left hand side of the figure) in the three main positions at the bifurcation [left arm (L), right arm (R) and main channel (M)]. At the right‐hand side, a merged figure with stained neutrophils (in green fluorescence) and endothelial cells‐impregnated microchannel (in contrast phase image) is shown

The migration activity of neutrophils was assayed with C5a treatment. Thus, although it did not reach the statistical analysis, the neutrophils from patients with obesity and HFpEF had a basal migratory activity major than neutrophils from the patient's group with obesity or HFpEF and those without obesity (1.15 ± 0.80 a.u. without obesity, 1.10 ± 0.88 a.u. obesity or HFpEF without obesity and 1.61 ± 0.45 a.u. basal/C5a in obesity and HFpEF) (Figure 3A).

### 3.4 CD11B upregulation in differentiated HL‐60 and adhesion

**FIGURE 4:**
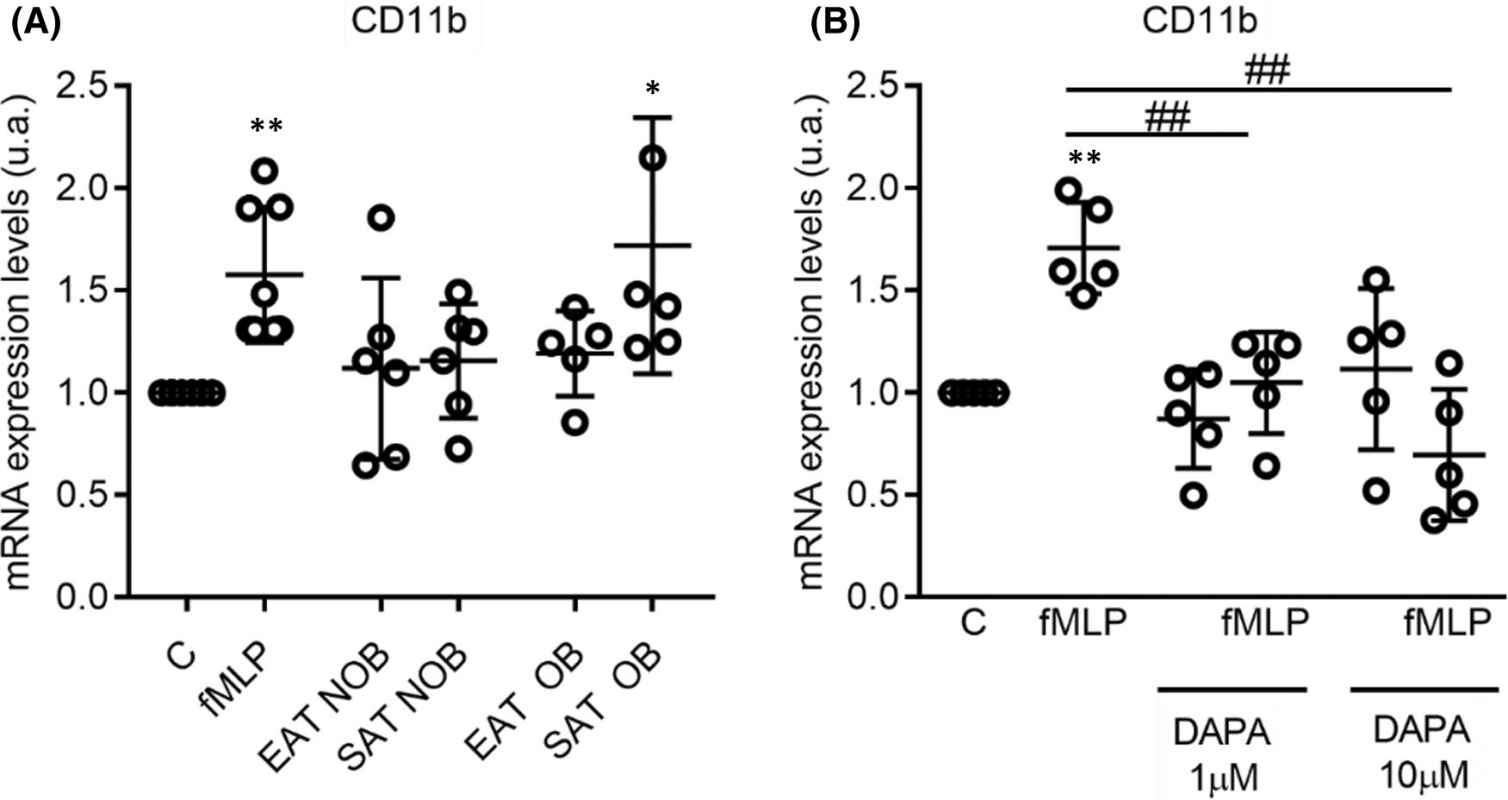
Released molecules of adipose tissue and mRNA CD11b expression levels in differentiated HL60 cells (A). Differentiated HL60 cells were treated with conditioned medium from subcutaneous (SAT) and epicardial (EAT) adipose tissue explants from patients without and with obesity. After performing a paired statistical test, CD11b mRNA expression levels were increased in treated cells with SAT‐conditioned medium from obese patients. Dot plots represents individual values and mean ± SD (*n* = 6) of CD11b mRNA levels in dHL‐60. Paired t‐test determined statistical differences between control and fMLP **p < .01, control and treatment with SAT‐conditioned medium from obese patients (SAT OB *p < .05). C = basal control, (cells in basal medium) and fMLP = positive control. Modulation of mRNA CD11b expression levels in differentiated HL60 cells by dapagliflozin (DAPA) (B). Differentiated HL60 cells were treated with dapagliflozin at different concentrations (1 and 10 μM) for 2 h before or not fMLP treatment. C = control, cells with basal medium, fMLP (cells with fMLP 1uM treatment), DAPA 1uM or 10 μM (cells with dapagliflozin pretreatment). Dot plots represents individual values and mean ± SD (*n* = 5) of CD11b mRNA levels in differentiated HL‐60. Paired t‐test determined statistical differences between control and fMLP **p < .01, fMLP and fMLP with DAPA 1 or 10 μM pretreatment ^##^ *p* < .01

Adhesion activity of neutrophils into endothelial cells after CD11B upregulation by fMLP was tested on a microfluidic assay (2.9 ± 0.6‐fold change; *p* < .05) (Figure 3B). Supernatants from epicardial and subcutaneous adipose tissue (EAT and SAT) of twelve patients were selected in a consecutive manner and matched regarding presence/absence of obesity. Clinical characteristics are described in Table S1. Differentiated HL‐60 were untreated (basal medium as control), treated or not with fMLP (positive control), conditioned medium from EAT and SAT from patients with or without obesity. Our results showed a significant increment of CD11B expression levels after fMLP stimulation (1.57 ± 0.33 a.u. over control) and conditioned medium of SAT from patients with obesity (1.71 ± 0.62 a.u. over control), *p* < .05). Although there is a slight upregulation of CD11B in differentiated HL‐60 after treatment with EAT‐supernatants from obesity (Figure 4A), we tested the SGLT2i, dapagliflozin, effects on CD11B modulation. Differentiated HL‐60 after fMLP stimulation with or without dapagliflozin pre‐treatment has shown a reduction of CD11B with dapagliflozin at 1 μM (1.70 ± 0.22 a.u. vs. 1.04 ± 0.24 a.u., ^##^ *p* < .01) and 10 μM (1.70 ± 0.22 a.u. vs. 0.69 ± 0.32 a.u., ^##^ *p* < .01) (Figure 4B).

## 4 DISCUSSION

Our results showed a differential neutrophils expression levels of CD11B, which can be regulated by adipose tissue‐released proteins, regarding obesity and emphasized in patients with HFpEF. The higher tyrosine kinase signalling identified by proteomics in neutrophils might confirm it because this signal transduction is associated with CD11B.^34^ Because this molecule is involved on transmigration through endothelial cells might suggest its role on inflamed endothelium, vasculature dysfunction and explain one of the mechanisms underlying HFpEF. Although further studies are needed, the neutrophils migration into heart can also contribute to myocardium hypertrophy, fibrosis and dysfunction.^35^ Moreover, since dapagliflozin reduces the anaerobic glycolysis,^36^ main metabolism on activated neutrophils, and CD11B expression levels, might suggest a protective role against inflammation response on patients with obesity and HFpEF progression.^37^ Moreover, the higher presence of RNase2 and 3 in neutrophils from obese patients, which are involved on extracellular matrix remodelling,^38^ might drive to an impaired myocardial regeneration.^38^, ^39^ Although further studies are needed, the results of this study contribute to understand the protective dapagliflozin mechanisms underlying HF.^40^

Obesity is a well‐known cardiovascular risk factor, characterized by higher white adipose tissue accumulation.^41^ High fat diet contributes to the neutrophils migration into adipose tissue^42^ and increase the pro‐inflammatory cytokines (tumour necrosis factor or monocyte chemoattractant protein 1) to promote monocytes recruitment.^43^, ^44^ The neutrophils elastase might participate in this process.^42^ Other described released mediators by neutrophils on obesity were IL‐8, MMP‐9 or MPO.^45^ The proteomic analysis has identified a differential proportion of involved proteins on tyrosine kinase signalling, adhesion or hormones between obese and non‐obese patients with cardiovascular disease. Thus, CD11B, which is upregulated in obesity and cardiovascular disease, is an integrin that participates in the leukocyte's adhesion activity. The higher tyrosine kinase signalling and lower hormone peptide in neutrophils from patients with obesity might suggest a higher participation of cytokines on CD11B upregulation and adhesion.^34^, ^46^ In fact, the reactome analysis has determined IL‐4 and IL‐13 signalling as the main upregulators of CD11B expression levels (Figure S2). Although some authors have described its upregulation in monocytes from patients with obesity and hypertension,^6^ we did not show it. The overlapping of both factors (obesity and HF) improved the increment of CD11B. This adhesion molecule can participate in the neutrophil's infiltration into myocardium and HF develop.^47^ Thus, we observed a higher basal migratory activity of neutrophils in those patients with obesity and HFpEF. Although C5a is a positive control of neutrophils migration activity, the presence of circulating chemokines or cytokines in these patients through tyrosine kinase signalling might explain their basal enhanced transmigration activity. Some authors have already described that determinant diastolic dysfunction in HFpEF is associated with higher MMP9 plasma levels^48^ that exacerbates the ischaemia‐induced chronic HF through the autophagy flux.^49^ However, we did not find differences of mRNA expression levels on circulating neutrophils. In the same line, our results did not show changes on neutrophil's MPO, although high plasma levels were associated with inflammation and neutrophils involvement in HFpEF.^50^ Similarly, plasma ICAM‐1 levels were associated with a worse prognosis on HFpEF^51^ but our neutrophils' transcriptional studies only detected changes regarding CD11B in an obesity and HFpEF phenotype. The early detection of neutrophils priming by transcriptomic changes might predict the cardiovascular disease progression and be useful for future therapeutical strategies.

Although RNase2 or RNase3 (ECP) was identified in 3 of the 9 obese patients, this molecule was not detected in neutrophils from patients without obesity. ECP is released by eosinophils and neutrophils.^52^ Although we did not purify neutrophils with specific antibodies, its mRNA expression levels were previously detected by other authors in mature neutrophils.^53^ This molecule might induce fibrosis because stimulates the secretion of tumour growth factor β (TGFβ), which is involved on fibroblasts proliferation.^38^ On the contrary, the regulation of this pathway might reduce the neutrophils infiltration on myocardium and ameliorates the cardiac remodelling in HFpEF.^54^ Other differential proteins expressed in neutrophils from patients with obesity and non‐obesity were the fetuin A and ceruloplasmin. This is mainly produced by liver and modulates the neutrophils oxidative status and apoptosis.^55^ This protein was identified in almost all patients except in 5 of them with obesity. In opposite, fetuin A enhances the apoptotic cells clearance.^56^ This protein appears in almost all patients except in 6 of them without obesity. In line with these finding, high fetuin A plasma levels were found in patients with obesity and metabolic disorders.^57^ However, proteomics studies cannot distinguish if the proteins are coming from cells or from a plasma contamination during the neutrophil's isolation protocol. Thus, changes on ceruloplasmin or fetuin A might be an artefact of plasma contamination. An important result of proteomics approach was the identification of RNase2 and RNase3 only in neutrophils from some of the obese patients with cardiovascular disease. The deposition of ECP on vascular lesions can stimulate platelets activity and accelerate thrombosis.^58^ This mechanism might explain the obesity‐related atherogenesis,^59^ although additional studies are needed to draw this conclusion.

Moreover, the contribution of released factors by SAT on CD11B transcription might suggest a systemic effect which could be modulated by dapagliflozin and protect against the subjacent inflammation to obesity and cardiovascular disease. Although further studies are required for understanding the mechanistic pathways involved on CD11B transcriptional regulation, these results suggest a possible dapagliflozin benefit on inflammation on HFpEF. The results of DELIVER trial will clarify these doubts.^60^

## 5 CONCLUSION

Our results have demonstrated that mRNA expression levels of CD11B and ECP are increased in neutrophils from patients with obesity and cardiovascular disease. The CD11B levels are pronounced in patients with obesity and HFpEF. White adipose tissue can contribute to this increment through its released proteins. The role of this integrin on endothelial adhesion and transmigration suggests new mechanisms involved on obesity and HFpEF which could be modulated by dapagliflozin.

## 6 LIMITATIONS

The isolation protocol for neutrophils does not allow delete basophils or eosinophils that are about 3%–5% of the total granulocytes. Although, the cellular population with a size between 9 and 12 μM was quantified by a Sceptre cell counter and used for the analysis, Polymorphprep allows isolate neutrophils with an 80% of purity. Proteomics analysis was performed with neutrophils lysed but we cannot distinguish if some of the proteins are coming from a plasma contamination. The assays lack of large animal models with HFpEF with and without obesity did not allow us to support the single contribution of obesity to CD11B upregulation as well was its modulation by dapagliflozin. Statistical analysis based on regressions was used for solving this experimental need.

## AUTHOR CONTRIBUTIONS

**Cristina Almengló:** Conceptualization (supporting); data curation (lead); formal analysis (lead); methodology (lead); writing – original draft (supporting). **Xiaoran Fu:** Data curation (supporting); methodology (supporting); writing – original draft (supporting). **María Teresa Flores‐Arias:** Data curation (supporting); formal analysis (supporting); methodology (supporting); writing – review and editing (supporting). **Ángel L. Fernández:** Data curation (supporting); investigation (supporting); methodology (supporting); writing – review and editing (supporting). **Juan E. Viñuela:** Data curation (supporting); methodology (supporting); writing – review and editing (supporting). **José M. Martínez‐Cereijo:** Data curation (supporting); methodology (supporting); writing – review and editing (supporting). **Darío Durán:** Data curation (supporting); methodology (supporting); writing – review and editing (supporting). **Moisés Rodríguez‐Mañero:** Data curation (supporting); methodology (supporting); writing – review and editing (supporting). **José R. González‐Juanatey:** Funding acquisition (lead); supervision (lead); writing – review and editing (supporting). **Sonia Eiras:** Conceptualization (lead); funding acquisition (lead); investigation (lead); project administration (lead); resources (lead); supervision (lead); validation (lead); visualization (lead); writing – original draft (lead); writing – review and editing (lead).

## CONFLICT OF INTEREST

Nothing to declare.

## Supporting information


Figure S1

Figure S2

Table S1

Table S2

## Data Availability

The data that support the findings of this study are available on request from the corresponding author. The data are not publicly available due to privacy or ethical restrictions.

## References

[1] Van Gaal LF , Mertens IL , De Block CE . Mechanisms linking obesity with cardiovascular disease. Nature. 2006;444:875‐880. 17167476 10.1038/nature05487

[2] Packer M . Epicardial adipose tissue may mediate deleterious effects of obesity and inflammation on the myocardium. J Am Coll Cardiol. 2018;71:2360‐2372. 29773163 10.1016/j.jacc.2018.03.509

[3] Yamashiro S , Kamohara H , Yoshimura T . MCP‐1 is selectively expressed in the late phase by cytokine‐stimulated human neutrophils: TNF‐α plays a role in maximal MCP‐1 mRNA expression. J Leukoc Biol. 1999;65:671‐679. 10331497 10.1002/jlb.65.5.671

[4] Witko‐Sarsat V , Rieu P , Descamps‐Latscha B , Lesavre P , Halbwachs‐Mecarelli L . Neutrophils: molecules, functions and pathophysiological aspects. Lab Invest. 2000;80:617‐654. 10830774 10.1038/labinvest.3780067

[5] De Filippo K , Rankin SM . CXCR4, the master regulator of neutrophil trafficking in homeostasis and disease. Eur J Clin Invest. 2018;48:e12949. 29734477 10.1111/eci.12949 PMC6767022

[6] Cui S , Qiao L , Yu S , et al. The antagonist of CXCR1 and CXCR2 protects db/db mice from metabolic diseases through modulating inflammation. Am J Physiol Endocrinol Metab. 2019;317:E1205‐E1217. 31573846 10.1152/ajpendo.00117.2019

[7] Friedrichs K , Adam M , Remane L , et al. Induction of atrial fibrillation by neutrophils critically depends on CD11b/CD18 integrins. PLoS One. 2014;9:e89307. 24558493 10.1371/journal.pone.0089307 PMC3928425

[8] Boschmann M , Engeli S , Adams F , et al. Adipose tissue metabolism and CD11b expression on monocytes in obese hypertensives. Hypertension. 2005;46:130‐136. 15976367 10.1161/01.HYP.0000171477.63859.b2

[9] Watanabe Y , Nagai Y , Takatsu K . Bidirectional crosstalk between neutrophils and adipocytes promotes adipose tissue inflammation. J Immunol. 2020;204:11821‐11835. 10.1096/fj.201900477RR 31355683

[10] Mahat RK , Singh N , Rathore V . Association of myeloperoxidase with cardiovascular disease risk factors in prediabetic subjects. Diabetes Metab Syndr Clin Res Rev. 2019;13:396‐400. 10.1016/j.dsx.2018.10.016 30641731

[11] Nijhuis J , Rensen SS , Slaats Y , van Dielen FMH , Buurman WA , Greve JWM . Neutrophil activation in morbid obesity, chronic activation of acute inflammation. Obesity. 2009;17:2014‐2018. 19390527 10.1038/oby.2009.113

[12] Cottam DR , Schaefer PA , Fahmy D , Shaftan GW , Angus LDG . The effect of obesity on neutrophil fc receptors and adhesion molecules (CD16, CD11b, CD62L). Obes Surg. 2002;12:230‐235. 11975218 10.1381/096089202762552674

[13] García‐Prieto CF , Gil‐Ortega M , Vega‐Martín E , et al. Beneficial effect of bariatric surgery on abnormal MMP‐9 and AMPK activities: potential markers of obesity‐related CV risk. Front Physiol. 2019;10:553. 31133882 10.3389/fphys.2019.00553 PMC6517546

[14] McDowell SAC , Luo RBE , Arabzadeh A , et al. Neutrophil oxidative stress mediates obesity‐associated vascular dysfunction and metastatic transmigration. Nat Cancer. 2021;2:545‐562. 35122017 10.1038/s43018-021-00194-9

[15] Li C , Zhang Z , Peng Y , et al. Plasma neutrophil gelatinase‐associated lipocalin levels are associated with the presence and severity of coronary heart disease. PLoS One. 2019;14:e0220841. 31387110 10.1371/journal.pone.0220841 PMC6684288

[16] Arbel Y , Finkelstein A , Halkin A , et al. Neutrophil/lymphocyte ratio is related to the severity of coronary artery disease and clinical outcome in patients undergoing angiography. Atherosclerosis. 2012;225:456‐460. 23040448 10.1016/j.atherosclerosis.2012.09.009

[17] Nakai ME , Denham J , Prestes PR , et al. Plasma lipocalin‐2/NGAL is stable over 12 weeks and is not modulated by exercise or dieting. Sci Rep. 2021;11:4056. 33603000 10.1038/s41598-021-83472-x PMC7893047

[18] Sabbah MS , Fayyaz AU , de Denus S , et al. Obese‐inflammatory phenotypes in heart failure with preserved ejection fraction. Circ Heart Fail. 2020;13:217‐228. 10.1161/CIRCHEARTFAILURE.119.006414 PMC7439286 32809874

[19] Sato T , Aizawa Y , Yuasa S , et al. The effect of dapagliflozin treatment on epicardial adipose tissue volume. Cardiovasc Diabetol. 2018;17:6. 29301516 10.1186/s12933-017-0658-8 PMC5753537

[20] Díaz‐Rodríguez E , Agra RM , Fernández ÁL , et al. Effects of dapagliflozin on human epicardial adipose tissue: modulation of insulin resistance, inflammatory chemokine production, and differentiation ability. Cardiovasc Res. 2018;114:336‐346. 29016744 10.1093/cvr/cvx186

[21] Byrne NJ , Matsumura N , Maayah ZH , et al. Empagliflozin blunts worsening cardiac dysfunction associated with reduced NLRP3 (nucleotide‐binding domain‐like receptor protein 3) Inflammasome activation in heart failure. Circ Heart Fail. 2020;13:e006277. 31957470 10.1161/CIRCHEARTFAILURE.119.006277

[22] Anker SD , Butler J , Filippatos G , et al. Empagliflozin in heart failure with a preserved ejection fraction. N Engl J Med. 2021;385:1451‐1461. 34449189 10.1056/NEJMoa2107038

[23] Zhang X , Wang T , Chen Z , et al. HMGB1‐promoted neutrophil extracellular traps contribute to cardiac diastolic dysfunction in mice. J Am Heart Assoc. 2022;11:e023800. 35156391 10.1161/JAHA.121.023800 PMC9245819

[24] Agra‐Bermejo RM , Cacho‐Antonio C , Rozados‐Luis A , et al. CD5L, macrophage apoptosis inhibitor, was identified in epicardial fat‐secretome and regulated by isoproterenol from patients with heart failure. Front Physiol. 2020;11:620. 32695009 10.3389/fphys.2020.00620 PMC7338428

[25] Shevchenko A , Wilm M , Vorm O , Mann M . Mass spectrometric sequencing of proteins from silver‐stained polyacrylamide gels. Anal Chem. 1996;68:850‐858. 8779443 10.1021/ac950914h

[26] Shilov IV , Seymour SL , Patel AA , et al. The paragon algorithm, a next generation search engine that uses sequence temperature values and feature probabilities to identify peptides from tandem mass spectra. Mol Cell Proteomics. 2007;6:1638‐1655. 17533153 10.1074/mcp.T600050-MCP200

[27] Tang WH , Shilov IV , Seymour SL . Nonlinear fitting method for determining local false discovery rates from decoy database searches. J Proteome Res. 2008;7:3661‐3667. 18700793 10.1021/pr070492f

[28] Denk S , Taylor RP , Wiegner R , et al. Complement C5a‐induced changes in neutrophil morphology during inflammation. Scand J Immunol. 2017;86:143‐155. 28671713 10.1111/sji.12580 PMC5773343

[29] Phieler J , Chung KJ , Chatzigeorgiou A , et al. The complement anaphylatoxin C5a receptor contributes to obese adipose tissue inflammation and insulin resistance. J Immunol. 2013;191:4367‐4374. 24043887 10.4049/jimmunol.1300038 PMC3817864

[30] Saito Y , Nakagawa C , Uchida H , Sasaki F , Sakakibara H . Adrenomedullin suppresses fMLP‐induced upregulation of CD11b of human neutrophils. Inflammation. 2001;25:197‐201. 11403211 10.1023/a:1011092532100

[31] Paradela‐Dobarro B , Rodiño‐Janeiro BK , Alonso J , et al. Key structural and functional differences between early and advanced glycation products. J Mol Endocrinol. 2016;56:23‐37. 26581238 10.1530/JME-15-0031

[32] Aymerich M , Álvarez E , Bao‐Varela C , Moscoso I , González‐Juanatey JR , Flores‐Arias MT . Laser technique for the fabrication of blood vessels‐like models for preclinical studies of pathologies under flow conditions. Biofabrication. 2017;9:025033. 28393759 10.1088/1758-5090/aa6c3d

[33] Schneider CA , Rasband WS , Eliceiri KW . NIH image to ImageJ: 25 years of image analysis. Nat Methods. 2012;9:671‐675. 22930834 10.1038/nmeth.2089 PMC5554542

[34] Futosi K , Mócsai A . Tyrosine kinase signaling pathways in neutrophils. Immunol Rev. 2016;273:121‐139. 27558332 10.1111/imr.12455

[35] Zhong Y , Yu X , Li X , Zhou H , Wang Y . Augmented early aged neutrophil infiltration contributes to late remodeling post myocardial infarction. Microvasc Res. 2022;139:104268. 34728225 10.1016/j.mvr.2021.104268

[36] Couselo‐Seijas M , Agra‐Bermejo RM , Fernández AL , et al. High released lactate by epicardial fat from coronary artery disease patients is reduced by dapagliflozin treatment. Atherosclerosis. 2020;292:60‐69. 31783199 10.1016/j.atherosclerosis.2019.11.016

[37] John JE , Claggett B , Skali H , et al. CAD is a risk factor for heart failure with preserved ejection fraction: the ARIC study. J Card Fail. 2019;25:S93.

[38] Zagai U , Dadfar E , Lundahl J , Venge P , Sköld CM . Eosinophil cationic protein stimulates TGF‐β1 release by human lung fibroblasts in vitro. Inflammation. 2007;30:153‐160. 17587163 10.1007/s10753-007-9032-4

[39] Wier E , Asada M , Wang G , et al. Neutrophil extracellular traps impair regeneration. J Cell Mol Med. 2021;25:10008‐10019. 34623736 10.1111/jcmm.16896 PMC8572775

[40] McMurray JJV , Solomon SD , Inzucchi SE , et al. Dapagliflozin in patients with heart failure and reduced ejection fraction. N Engl J Med. 2019;381:1995‐2008. doi:10.1056/nejmoa1911303 31535829

[41] Jia G , Jia Y , Sowers JR . Contribution of maladaptive adipose tissue expansion to development of cardiovascular disease. Compr Physiol. 2017;7:253‐262. 10.1002/cphy.c160014 28135006

[42] Talukdar S , Oh DY , Bandyopadhyay G , et al. Neutrophils mediate insulin resistance in mice fed a high‐fat diet through secreted elastase. Nat Med. 2012;18:1407‐1412. 22863787 10.1038/nm.2885 PMC3491143

[43] Trim W , Turner JE , Thompson D . Parallels in immunometabolic adipose tissue dysfunction with ageing and obesity. Front Immunol. 2018;9:169. 29479350 10.3389/fimmu.2018.00169 PMC5811473

[44] Kane H , Lynch L . Innate immune control of adipose tissue homeostasis. Trends Immunol. 2019;40:857‐872. 31399336 10.1016/j.it.2019.07.006

[45] Rouault C , Pellegrinelli V , Schilch R , et al. Roles of chemokine ligand‐2 (CXCL2) and neutrophils in influencing endothelial cell function and inflammation of human adipose tissue. Endocrinology. 2013;154:1069‐1079. 23372021 10.1210/en.2012-1415

[46] Sindhu S , Thomas R , Shihab P , Sriraman D , Behbehani K , Ahmad R . Obesity is a positive modulator of IL‐6R and IL‐6 expression in the subcutaneous adipose tissue: significance for metabolic inflammation. PLoS One. 2015;10:e0133494. 26200663 10.1371/journal.pone.0133494 PMC4511728

[47] Maier HJ , Schips TG , Wietelmann A , et al. Cardiomyocyte‐specific IκB kinase (IKK)/NF‐κB activation induces reversible inflammatory cardiomyopathy and heart failure. Proc Natl Acad Sci U S A. 2012;109:11794‐11799. 22753500 10.1073/pnas.1116584109 PMC3406816

[48] Martos R , Baugh J , Ledwidge M , et al. Diagnosis of heart failure with preserved ejection fraction: improved accuracy with the use of markers of collagen turnover. Eur J Heart Fail. 2009;11:191‐197. 19168518 10.1093/eurjhf/hfn036 PMC2639413

[49] Nandi SS , Katsurada K , Sharma NM , Anderson DR , Mahata SK , Patel KP . Mmp9 inhibition increases autophagic flux in chronic heart failure. Am J Physiol Hear Circ Physiol. 2020;319:1414‐1437. 10.1152/ajpheart.00032.2020 PMC7792705 33064567

[50] Hage C , Michaëlsson E , Kull B , et al. Myeloperoxidase and related biomarkers are suggestive footprints of endothelial microvascular inflammation in HFpEF patients. ESC Hear Fail. 2020;7:1534‐1546. 10.1002/ehf2.12700 PMC7373930 32424988

[51] Kanagala P , Arnold JR , Khan JN , et al. Plasma P‐selectin is a predictor of mortality in heart failure with preserved ejection fraction. ESC Hear Fail. 2021;8:2328‐2333. 10.1002/ehf2.13280 PMC8120355 33694306

[52] Sur S , Glitz DG , Kita H , et al. Localization of eosinophil‐derived neurotoxin and eosinophil cationic protein in neutrophilic leukocytes. J Leukoc Biol. 1998;63:715‐722. 9620664 10.1002/jlb.63.6.715

[53] Monteseirín J , Vega A , Chacón P , et al. Neutrophils as a novel source of eosinophil cationic protein in IgE‐mediated processes. J Immunol. 2007;179:2634‐2641. 17675527 10.4049/jimmunol.179.4.2634

[54] Zhang L , Chen J , Yan L , He Q , Xie H , Chen M . Resveratrol ameliorates cardiac remodeling in a murine model of heart failure with preserved ejection fraction. Front Pharmacol. 2021;12:646240. 34177571 10.3389/fphar.2021.646240 PMC8225267

[55] Golenkina E , Viryasova G , Galkina S , Gaponova T , Sud’ina G , Sokolov A . Fine regulation of neutrophil oxidative status and apoptosis by ceruloplasmin and its derivatives. Cell. 2018;7:8. 10.3390/cells7010008 PMC5789281 29329239

[56] Jersmann HPA , Dransfield I , Hart SP . Fetuin/α2‐HS glycoprotein enhances phagocytosis of apoptotic cells and macropinocytosis by human macrophages. Clin Sci. 2003;105:273‐278. 10.1042/CS20030126 12725640

[57] Trepanowski JF , Mey J , Varady KA . Fetuin‐a: a novel link between obesity and related complications. Int J Obes (Lond). 2015;39:734‐741. 25468829 10.1038/ijo.2014.203

[58] Hallgren R , Gudbjornsson B , Larsson E , Fredens K . Deposition of eosinophil cationic protein in vascular lesions in temporal arteritis. Ann Rheum Dis. 1991;50:946‐949. 1768167 10.1136/ard.50.12.946 PMC1004589

[59] Murphy AJ , Tall AR . Disordered haematopoiesis and athero‐thrombosis. Eur Heart J. 2016;37:1113‐1121. 26869607 10.1093/eurheartj/ehv718 PMC4823636

[60] Solomon SD , de Boer RA , DeMets D , et al. Dapagliflozin in heart failure with preserved and mildly reduced ejection fraction: rationale and design of the DELIVER trial. Eur J Heart Fail. 2021;23:1217‐1225. doi:10.1002/EJHF.2249 34051124 PMC8361994

