# Supplementary material for: Synergism between obesity and HFpEF on neutrophils phenotype and its regulation by adipose tissue‐molecules and SGLT2i dapagliflozin
Source: J Cell Mol Med. 2022 Jul 11;26(16):4416–27. doi: 10.1111/jcmm.17466 (PMC9357605; doi:10.1111/jcmm.17466)
Supplement: Supplementary file 1 — Figure S1 [file JCMM-26-4416-s001.ppt]

## Slide 1
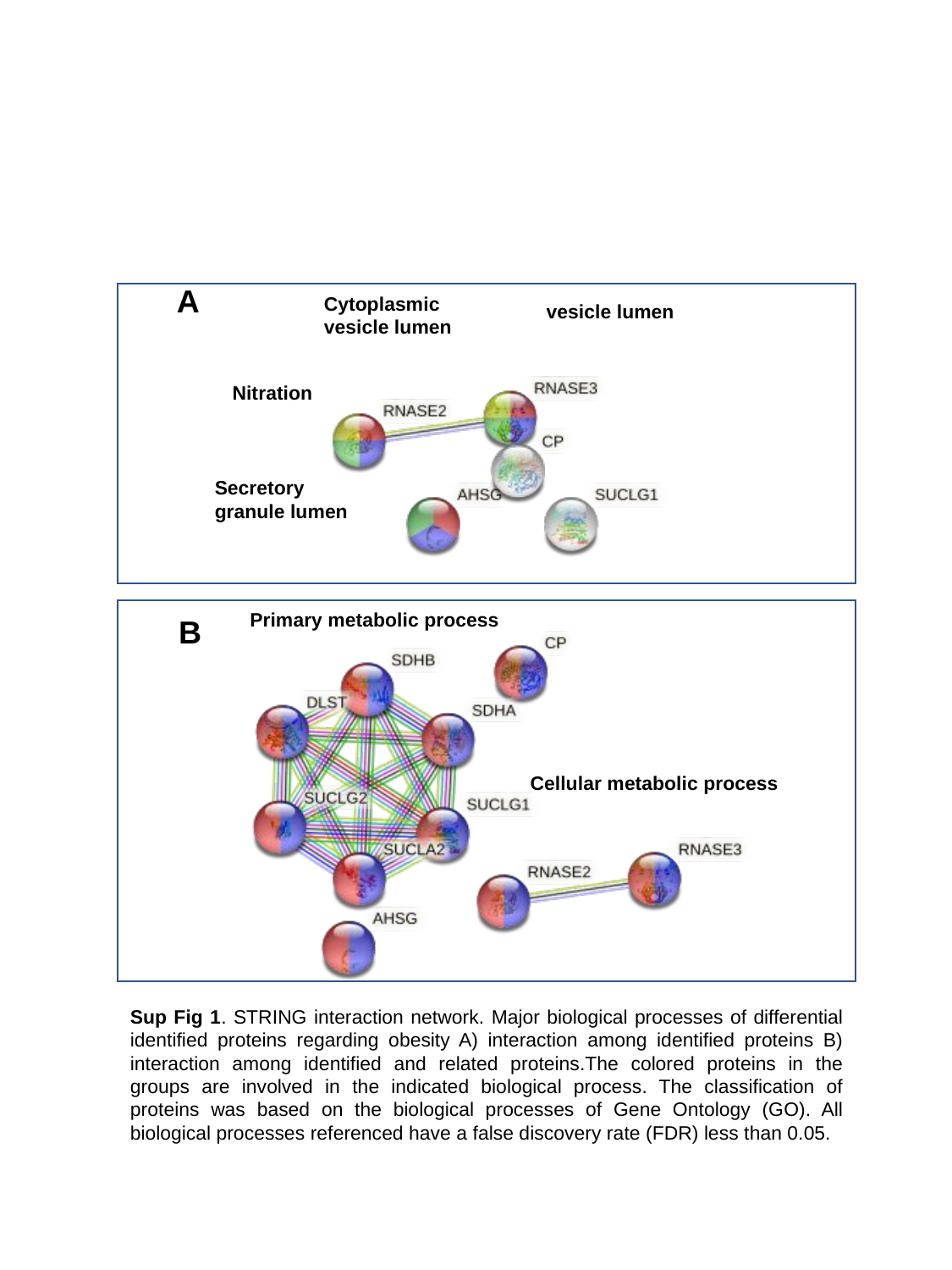

A
Cytoplasmic vesicle lumen
vesicle lumen
Nitration
Secretory granule lumen
Primary metabolic process
Cellular metabolic process
B
Sup Fig 1. STRING interaction network. Major biological processes of differential identified proteins regarding obesity A) interaction among identified proteins B) interaction among identified and related proteins.The colored proteins in the groups are involved in the indicated biological process. The classification of proteins was based on the biological processes of Gene Ontology (GO). All biological processes referenced have a false discovery rate (FDR) less than 0.05.

## Slide 2
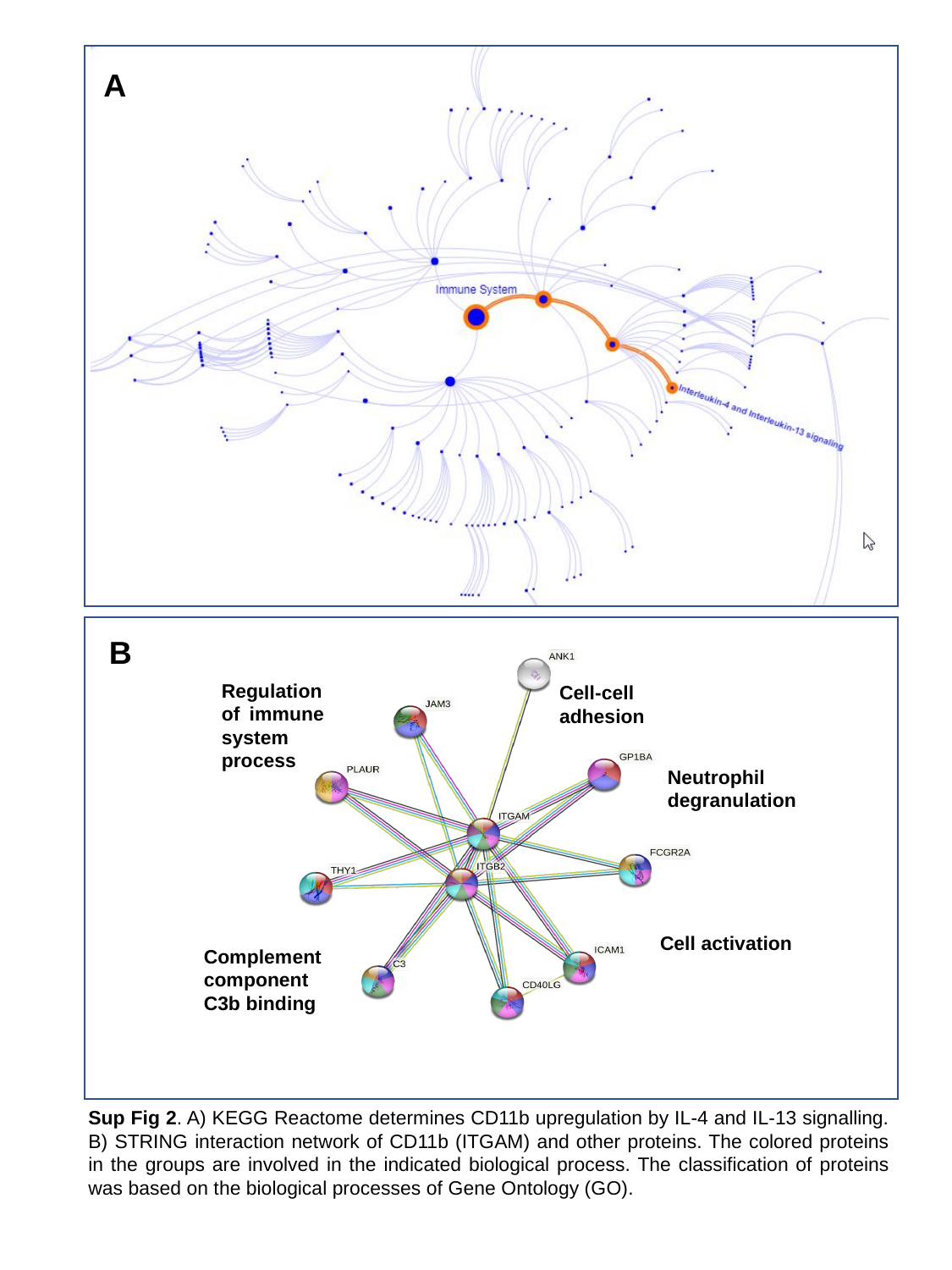

A
B
Regulation of immune system process
Cell-cell adhesion
Neutrophil degranulation
Cell activation
Complement component C3b binding
Sup Fig 2. A) KEGG Reactome determines CD11b upregulation by IL-4 and IL-13 signalling. B) STRING interaction network of CD11b (ITGAM) and other proteins. The colored proteins in the groups are involved in the indicated biological process. The classification of proteins was based on the biological processes of Gene Ontology (GO).
