# Supplementary material for: Synergism between obesity and HFpEF on neutrophils phenotype and its regulation by adipose tissue‐molecules and SGLT2i dapagliflozin
Source: J Cell Mol Med. 2022 Jul 11;26(16):4416–27. doi: 10.1111/jcmm.17466 (PMC9357605; doi:10.1111/jcmm.17466)
Supplement: Supplementary file 3 — Table S1 [file JCMM-26-4416-s004.docx]

|  | NOB | OB | *p* |
| --- | --- | --- | --- |
| N | 10 | 10 | - |
| BMI (kg/m²) | 26,4 (2,7) | 33,37(6,3) | **0,005** |
| Age | 67,90 (7,8) | 68,90 (5,3) | 0,372 |
| Gender (female/male) | 2/8 | 2/8 | 1,000 |
| CAD (no/yes) | 5/5 | 3/7 | 0,361 |
| T2DM (no/yes) | 5/5 | 6/4 | 0,653 |
| AHT (no/yes) | 7/3 | 6/4 | 0,639 |
| DLP (no/yes) | 0/10 | 3/7 | 0,060 |
| KF (no/yes) | 10/0 | 9/1 | 0,305 |
| HF (no/yes) | 3/7 | 4/6 | 0,639 |
| AF (no/yes) | 5/5 | 5/5 | 1,000 |

**Supplementary table 1**. **Clinical characteristics of patients according obesity presence for neutrophils´ s proteomics studies (A) or adipose tissue explants assays (B)**. Unpaired t-test was used for continues variables differences and Person X2 for categorical variables. Statistical significance p<0.05. LVEF: Left Ventricular Ejection Fraction; T2DM: Type 2 diabetes mellitus, CAD: Coronary artery disease; HF: Heart Failure; DLP: Dyslipidemia; AHT: Arterial Hypertension.

**A**

|  | NOB | | OB | | *p* |
| --- | --- | --- | --- | --- | --- |
| N | | 6 | | 6 | - |
| BMI (kg/m²) | | 25,5 (2,3) | 32,3 (2,1) | | **0,016** |
| Age | | 71,16 (12,9) | 74,66 (9,4) | | 0,604 |
| Gender (female/male) | | 2/4 | 1/5 | | 0,505 |
| CAD (no/yes) | | 4/2 | 4/2 | | 1,000 |
| T2DM (no/yes) | | 2/4 | 3/3 | | 0,558 |
| AHT (no/yes) | | 3/3 | 1/5 | | 0,221 |
| DLP (no/yes) | | 3/3 | 1/5 | | 0,221 |
| HF (no/yes) | | 5/1 | 2/4 | | 0,079 |
| AF (no/yes) | | 3/3 | 1/5 | | 0,221 |

**B**
