# Supplementary material for: Synergism between obesity and HFpEF on neutrophils phenotype and its regulation by adipose tissue‐molecules and SGLT2i dapagliflozin
Source: J Cell Mol Med. 2022 Jul 11;26(16):4416–27. doi: 10.1111/jcmm.17466 (PMC9357605; doi:10.1111/jcmm.17466)
Supplement: Supplementary file 4 — Table S2 [file JCMM-26-4416-s002.docx]

**Supplementary table 2**. Differential neutrophils-proteins between groups with and without obesity

SUCA

ECP

CERU

RNAS2

FETUAAA
